# Polyetheretherketone Material in Dentistry

**DOI:** 10.7759/cureus.46485

**Published:** 2023-10-04

**Authors:** Kshitija P Parate, Naleen Naranje, Rozina Vishnani, Priyanka Paul

**Affiliations:** 1 Public Health Dentistry, Sharad Pawar Dental College and Hospital, Datta Meghe Institute of Higher Education and Research, Wardha, IND; 2 Oral and Maxillofacial Surgery, Sharad Pawar Dental College and Hospital, Datta Meghe Institute of Higher Education and Research, Wardha, IND

**Keywords:** aesthetic, abutment, prosthesis, implant, peek

## Abstract

A polyaromatic nearly-crystalline thermoplastic polymer, polyetheretherketone (PEEK), has become a useful biomaterial and its use has increased in dentistry because of its properties. PEEK is scientifically approved and is among the safest material used to restore lost orofacial tissues at present. PEEK has a property of high biocompatibility, therefore there is increased utilization of PEEK in orthopaedic and trauma cases. PEEK has several excellent properties due to which it has been used in several fields of dentistry such as orthodontic wires, implants, removable dentures, fixed partial dentures, finger prostheses, temporary abutments, implant-supported provisional crowns, healing caps, maxillofacial prostheses, etc. Due to its modification, PEEK material is used more frequently in clinical dentistry. PEEK can be used as a material that is not traditional in the realm of dental care. Modification of PEEK has led to an increase in its use in the field of dentistry.

## Introduction and background

This article introduces polyetheretherketone (PEEK) as a promising material for dental restorations. Alloy-free restorations are becoming more common in current dental procedures because of cosmetic concerns. PEEK is a member of the polyaryletherketone (PAEK) family. The combination of three functional groups results in PAEK, a material with great resistance to attack by chemicals, beneficial toughness, and high strength, along with heat resistance and good processability because the phenylene rings are non-reactive, the ether group offers flexibility, and the ketone group rigidity. PEEK is one of the various metal-free restorations that could be used in dentistry. Given its superior qualities in prosthodontics and oral implantology, PEEK is becoming more significant [1]. Branemark introduced titanium and its alloy as dental implants, but titanium materials have certain disadvantages such as allergies, surface degradation, and hypersensitivity reactions [2]. Apart from this, because of its metallic color, it cannot be used in aesthetic regions [2]. PEEK is a material with radiolucence while titanium is radio-opaque. Excellent aesthetic and mechanical qualities can be found in PEEK than titanium [2]. PEEK has unique physical qualities and is a biocompatible material; hence, it can be used in patients who are allergic to metallic components, while titanium shows allergic reaction but in very rare cases [2]. Both fixed and removable dental prostheses and orthodontic wires can be supported by PEEK because of its excellent mechanical and aesthetic attributes [2,3]. The PEEK material is obtained from functional groups that mainly bind ketone and ether with acyl rings. PEEK has ultrahigh performance and chemical resistance. PEEK may be used as a bone alternative for dental implants, osteosynthesis plates, cranial implants, and reconstructions of the nose, maxilla, and mandible. The modulus of elasticity of PEEK is very close to that of bone; therefore, it is used as an implant material. PEEK provides radiolucent imaging and is compatible with various imaging techniques such as X-ray, magnetic resonance imaging, and computed tomography. PEEK material can be incorporated with carbon fiber or glass and can be used with nanomaterial to enhance the properties of PEEK. A stress-dispersion mode identical to that of natural teeth may be achieved via the shock-absorbing action offered by PEEK during occlusion and chewing force. Bacteria or microorganisms must not be permitted to cling to the outer layer of dental materials used as implant abutments as doing so leads to the creation of biofilms around the implant and abutment, which eventually end up in peri-implantitis and peri-implant mucositis. This unwanted reaction not only stresses the patient psychologically but also increases the risk of early implant failure. According to some research, PEEK does not form a biofilm on its surface and can be used as an implant material [4]. Compared with conventional implant-abutment materials, PEEK exhibits cell metabolic activity, attachment of cells, and pro-inflammatory cytokine responses when exposed to human oral fibroblasts. PEEK also shows hypersensitivity reactions such as allergy, swelling, itching, urticaria, and erythema, but it is very rare. PEEK has long been subject to plasma changes. Increased adhesion, proliferation, and osteogenic differentiation were observed as a result of this alteration. As a substitute for stainless steel bone plates, carbon fiber reinforced (CFR)-PEEK fixation plates have been created. Typically, metals, ceramics, composites, and polymers have applications in orthopedic implants. Permanent and temporary implants are made of metals such as nickel-titanium (Ni-Ti), titanium, and cobalt-chromium (Co-Cr), although these materials have limitations. Allergies, a high elastic modulus that can stress the peri-implant bone, and the radiopacity of this metal that generates artifacts in CT scans are some of the disadvantages of metal [2]. Hence, PEEK is considered a better material than other metallic materials. PEEK materials are cemented using dual-cure resin cement. The low surface energy of the PEEK material is one of its important drawbacks when used in prosthetic dentistry. PEEK demonstrated poor adhesion to the resin cement. The high flexibility modulus of metal substructures and the negative stress concentration in the cement interface, which causes abutment tooth movement, are two of the primary causes of cement bonding failure. The PEEK surface energy is enhanced by utilizing conventional sanding, acid roughening, plasma spray, and laser roughening techniques According to certain researchers, veneer coating is required because PEEK does not have sufficient clarity. PEEK can replace metal alloys and zirconium dioxide as a substructure material because of its color and high mechanical qualities. The veneer coating process improves the appearance.

## Review

Search methodology

We conducted the review through PubMed and Google Scholar in July 2022 using keywords such as "PEEK", "implant", "prosthesis", "aesthetic" and "abutment". In addition, we looked through the bibliographies of pertinent studies for important references. February 2023 witnessed an update to the search. The reviewer first checked the titles and abstracts of the retrieved papers against the inclusion and exclusion criteria before checking the complete texts. Both published and unpublished English-language research were taken into consideration for inclusion. Due to resource constraints and the reviewer's inability to access full-text articles, we omitted research and articles that are related to use of PEEK in other fields like non-biomedical applications.

Properties and structure of polyetheretherketone

**Figure 1:**
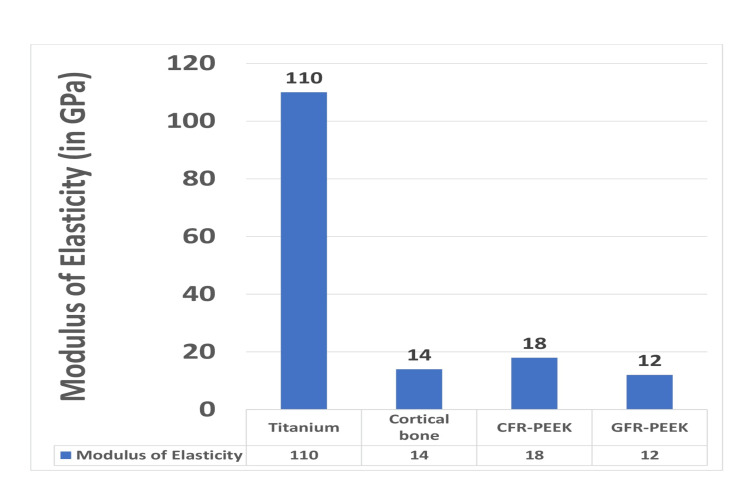
Modulus of Elasticity Created by author with reference to data from source [2] CFR-PEEK: carbon fiber reinforced polyetheretherketone, GFR-PEEK: glass-fiber reinforced polyetheretherketone

PEEK belongs to the PEAK family of superior-performance polymers. PEEK is a monochromatic, thermoplastic, semicrystalline polymer. It is considered the most important member of the PEAK family [5]. It is a homopolymer made of only one type of monomer. It has superior chemical and mechanical properties such that it can withstand higher temperatures. PEEK shows resistance to corrosion during the sterilization procedure. Due to this property, it can be sterilized using a heat sterilization process. It has high strength property, thermal property, and high strength high modulus of elasticity. The modulus of elasticity of carbon fibre-reinforced PEEK and glass fibre-reinforced PEEK is close to that of the bone as shown in Figure 1 [2], therefore there is homogenous distribution of stress to surrounding tissues. It is highly biocompatible with surrounding tissues.

Numerous studies have looked into PEEK's ability to behave as a comparatively bio-inactive polymer with little fixation to bone. The amount of bone resorption surrounding the implant fixture and implant stiffness are correlated. It is not cytotoxic, mutagenic, carcinogenic, or immunogenic to surrounding structures [6]. PEEK has a low-density and lightweight material. It has a low modulus of elasticity which is almost close to the bone. PEEK does not have a metallic color therefore it has superior aesthetic properties. This material can be produced using computer-aided design/computer-aided manufacturing (CAD/CAM) technology. PEEK has wear resistance that is similar to that of metal alloys while having a somewhat poor elastic strength and low hardness. In contrast to resin materials, PEEK demonstrates higher wear resistance when applying lateral force and a similar rate of abrasion. Among other plastic substitutes, PEEK demonstrated the greatest flexural strength and creep resistance. PEEK is more soluble in water and is not chemically impacted by prolonged exposure to water, even at higher temperatures.

PEEK consists of aromatic rings of ketone and ether functional groups [7]. The structure of PEEK is highly resistant to radiation and chemical destruction. It is compatible with agents such as carbon and glass fibers and has greater strength than metals [8]. At high temperatures PEEK implant is stable because of its length, biochemical make-up, and architecture (polymer branching) that allow for simple production of PEEK implant parts. There is very little effect on PEEK after exposure to chemicals. PEEK also has the property of moisture absorption [9].

Uses of polyetheretherketone in dentistry

Unlike metal, PEEK is biocompatible and has a natural tooth-colored look, hence PEEK's extensive usage in implants, provisional abutments, implant-supported bars, or clamp materials in the production of removable dental prostheses (RDP), fixed prostheses and maxillary obturator prostheses. Due to enhanced aesthetics, allergies to metals, and interference of metals with thermal conductivity (TC), PEEK also provides an alternative to orthodontic wires. Additionally, PEEK is easily moulded with dental burs despite still requiring veneering because of its poor translucency and greyish colour.

Due to the surface modification of PEEK, it has allowed it to attach to different luting agents. Its tensile properties are very similar to bone, enamel, and dentin due to which it can be used as a dental restorative material. PEEK has become widespread in the dental field and competitive level with titanium material.

PEEK in Dental Implants

Branemark has introduced titanium as dental implants and it has become the material of choice for dental implants. But titanium has certain disadvantages such as allergy and hypersensitivity reactions in very rare cases [2]. The mouldability of titanium and osseous materials is different which leads to bone resorption and stress shielding [10]. A major feat of PEEK implants is that Young's modulus is almost similar for PEEK material and bone, which prevents bone resorption. Titanium has a metallic color and lacks light transmission properties, hence it has an unesthetic appearance and cannot be used where esthetics are indicated. Thus, PEEK can be an alternative to titanium [2]. Surface modifications of PEEK have been created to boost cellular reaction. 

PEEK is bio inactive material with limited biological properties such as limited bone fixation so it's bioactivity can be increased by contacting medication such as plasma modification. Plasma alterations produced osteogenic differentiation, proliferation, and adhesion [10]. Chemical processes like sulfonation and titanium surface coating dioxide and gold have been used. Studies have been made on hydroxyapatite (HA) coating, to enhance the cell attachment on the surface of PEEK implant. Excellent results have been found from PEEK implants coated with HA than non-coated PEEK implants [11].

Different tissue activity and cytokines associated with inflammation around various implant materials were discovered by histological analyses. Alkaline phosphatase and osteocalcin levels are greater near titanium-aluminum-vanadium alloys but DNA is less noticeable in the area around PEEK implants. However, it has been found that levels of the proinflammatory cytokines IL1-ß, IL-6, and IL-8 are higher around PEEK implants and, at the very least, titanium alloys. In comparison to PEEK, titanium alloys have higher levels of the anti-inflammatory cytokine IL-10. The production of these proinflammatory cytokines is what leads to the creation of fibrosis tissue around PEEK implants. Titanium alloy surfaces, however, offer a more hospitable environment for osteogenic activity.

PEEK as Abutments

Various materials have been used in the production of abutments such as titanium, zirconia, ceramics, and gold. But titanium has certain disadvantages such as corrosion leading to over-sensitivity reactions [12]. But in cases of aesthetics as a priority, the conclusions fall short of satisfying. Zirconia was used as an abutment, but due to its corrosive property and poor mechanical properties, the use of zirconia is restricted when compared to single-tooth implants with whole ceramic prostheses. The temporary crown on the PEEK abutment and the titanium temporary abutment have the same fracture strength. As fracture resistance of unmodified PEEK is lower than titanium it cannot be used as a definite abutment material. Unmodified PEEK can be used as a provisional abutment. It reduces the stress shielding effect around the implant [13].

PEEK has high mechanical properties; it can be used as both abutment and prosthetic material. As the PEEK material's elastic quality lessens the pressures generated when chewing and transmitted It has been asserted that forces occurring in the tooth abutment also at the cement contact are exaggerated concerning the implant because of the low elastic modulus of this component minimized [14]. PEEK can replace the titanium material in abutment production because it has the property of bone remodeling. According to Hendrik's study, the application of a composite resin crown on titanium and PEEK material, the crown was placed across the low-resistance PEEK foundations [15]. In some studies zirconium and PEEK abutment were compared, no breakage was observed in both the material but the deformation was observed in PEEK abutment. PEEK reduces fragility and it deforms but does not break. Intraorally, zirconium abutments deteriorate over time. Additionally, the internal structure changes as a result of the poor mechanical resistance. Disadvantages of this substance include degradation in water and aqueous solutions, deterioration at low temperatures, and transformation from a tetragonal phase to a monoclinic phase. If we consider a situation like screw breakage, PEEK can be removed more easily than other materials.

PEEK in Fixed Prosthesis

PEEK can be veneered with composites because of its opacity to achieve aesthetics. By adding the proper pigments, it is possible to alter PEEK's grey color in unfilled material. PEEK is chemically inert, hence many techniques have been tried to achieve a strong bond with veneering materials. Sandblasting, the locate process, surface etching with sulfuric acid, and piranha solution are some of these surface treatments. Plasma surface modification is a more recent technique that allows for surface activation and cross-linking as well as the removal of organic residues. PEEK has had its surface modified to bind with various luting agents [16]. Multifunctional methacrylates contain resin varnish on air-abraded PEEK surfaces generating a promising and long-lasting bond to PEEK, making PEEK suitable for usage in clinical settings [17,18].

PEEK in Removable Prosthesis

PEEK can be utilized to make clasps and dentures using CAD/CAM systems because of its low weight and high biological, cosmetic, and mechanical qualities. Making removable obturators is an additional use. The palatal section of maxillary obturator prostheses can use PEEK-OPTIMA (reinforced poly-ether-ether ketone) due to its biological compatibility, durability, torsional bone modulus, machinability, and processing simplicity [19].

"The use of PEEK eased the manufacture of the antral component of the maxillary obturator prosthesis", according to Costa-Palau et al., "and produced a substantially lighter prosthesis. Retention, appearance, and patient comfort all saw significant improvements." [20]. For patients with significant oral-nasal abnormalities, using PEEK-OPTIMA to make obturator prostheses is a good substitute for traditional materials and techniques. Because of PEEK's strength and lightweight, a digital design that adapts to each patient's anatomy, lack of heat and electric conductivity, compatibility with scanners as well as x-rays, and non-allergenic qualities, patient comfort is increased.

PEEK frameworks provide remarkable resistance to deterioration and abrasion in addition to being shock-absorbing during mastication. Even though metals have considerable strength, patient comfort, and resilience are also major concerns. Despite having strong fracture resistance, homogenous PEEK has poor mechanical properties. According to in vitro studies by Tannous et al., "PEEK clasps exhibit lower resistance forces than cobalt chrome." [21]. This resulted in the creation of a modified PEEK known as BioHPP (Bredent GmbH, Senden, Germany) that contains 19.97% ceramic fillers. High-quality prostheses are produced using BioHPP due to their capacity to be corrected, good stability, ideal polishing ability, and aesthetics. In patients with high aesthetic demands, BioHPP, which has enormous promise as a framework material, is a promising alternative to Cr-Co frameworks [22].

PEEK has high resistance and PEEK is white therefore it can be used in the preparation of metal hooks and braces [23]. PEEK can be used as implant-supported bars [24]. PEEK and acrylic teeth can be used together as removable partial prosthesis material [25]. PEEK is an overtime restoration method as it has low solubility and low water absorption [26,27].

PEEK in Orthodontics

Orthodontic wire made of PEEK material has higher strength and appears aesthetic. PEEK is nonmetallic and aesthetic and it has high strength and high resistance to breakage [28,29]. PEEK can also be used as a space maintainer. PEEK has higher creep resistance and optimal properties so it is used as orthodontic wires [30]. Due to its high bending capacity, creep resistance, similar orthodontic force to Ni-T wire, and low water absorption, PEEK is regarded as a nonmetal and cosmetic arc wire. The metal arch wire was run through the PEEK tube in the Shirakawa et al. PEEK tube method to produce an acceptable aesthetic appearance. Ni-Ti wires can be replaced with PEEK wires in some applications.

Advantages and disadvantages of PEEK are shown in Table 1 [31-33].

**Table 1:** Advantages and disadvantages of PEEK PEEK: polyetheretherketone

Advantage	Disadvantage
Resistance to degradation	It requires a high processing temperature.
Antiallergic	It is very expensive.
Resistant to chemicals	PEEK can degrade at temperature between glass transition and melt transition, it can degrade in the body.
An elastic modulus comparable to bone	PEEK has low surface energy due to which it has less cell adhesion.
Carbon fibre can be reinforced with PEEK	Difficulty in manufacturing.

Comparison of polyetheretherketone with other material

It has been established that using titanium in implants is associated with a number of disadvantages. These include user hypersensitivity, excessive tension placed on the implant-bone as a result of the gradient of different elastic moduli, and specific aesthetic issues. Titanium has been suggested as a replacement for PEEK, which was first commercialized as a biomaterial for implants.

Since PEEK abutments lessen stress shielding between dental implants and the nearby alveolar bone, they are also accessible as abutments for temporary implant restorations. PEEK abutments have just recently been introduced to the field of implant dentistry. It was demonstrated using PEEK as a temporary abutment that this polymer offers suitable labial/buccal shapes and support for the papillary tissues. The PEEK abutment is also reasonably priced, adaptable to support a temporary prosthesis at the time of implant implantation, and easier to create a good provisional aesthetic outcome due to its color. In contrast to titanium temporary abutments, which are advised for longer periods in the mouth, PEEK provisional abutments demonstrated less fracture resistance than titanium abutments. As a result, PEEK abutments are recommended for placement of provisional fixed prostheses for a shorter period of time.

Metal restorations are heavily weighted as compared to PEEK materials. Intraoral repairing can be possible in PEEK material. PEEK exhibits low brittle behavior and has more stress-absorbing effect than ceramic materials [34]. For the creation of metal-free restorations, PEEK is a biologic, high-impact polymer material, lowering the risk of an allergic reaction or any corrosion phenomenon. Compared to metal alloys, it has a lower specific weight, which makes restorations lighter. PEEK is the sole material used to fabricate ceramic frameworks that has a stress-absorbing effect and a less brittle behavior. Its use in implant restorations where the periodontal ligament is absent may also be indicated by a similarity to the bone's modulus of elasticity. PEEK is widely utilized in orthopaedics and has increased biocompatibility. The low plaque buildup and low moisture absorption may support its usage for intraoral use. However, whether the material's surface polish can be compared to glazed ceramic or parts made of titanium in factories is debatable. When compared to ceramic materials, PEEK frameworks can be produced more cheaply using traditional methods or CAD/CAM technologies. Several techniques, including direct milling of PEEK blanks or 3D printing of a resin/wax pattern framework that is then thermopressed via the traditional lost-wax/resin procedure, can be used to create CAD-CAM PEEK RDP frameworks [35]. The fabrication process is further made simpler by using light-polymerizing polymer veneering materials, which saves time and money. The ability to intra-oral replace the veneering material in cases of chipping without removing the restoration is another significant therapeutic benefit of PEEK restorations. The fatigue strength of PEEK frameworks is a clinical concern that hasn't been looked at because there aren't enough in-vitro experiments. Both in-vitro and in-vivo studies are required to verify the durability of the link between polymer veneering materials and PEEK frameworks. Finally, before widespread therapeutic usage of this novel material is advised, the overall clinical performance of PEEK needs to be examined in lengthy clinical trials.

## Conclusions

PEEK has excellent physical, mechanical, aesthetic, and biocompatibility properties therefore PEEK can be used in a variety of dental uses, comprising implants, orthodontic wires, fixed and temporary prostheses. PEEK can withstand in higher temperatures and has high resistance therefore can be used in dentistry. PEEK material can be incorporated with carbon fiber or glass and can be used with nanomaterial to enhance the properties of PEEK. The key challenge for PEEK as an implant material is increasing bioactivity while keeping the material's properties. PEEK's bioactivity using various tools and materials, more research is still needed. PEEK may be used as a bone substitute for dental implants, osteosynthesis plates, cranial implants, and reconstructions of the nose, maxilla, and mandible. Manufacturing of customized prostheses has been made possible through CAD/CAM and fast prototyping. Carbon fiber reinforcement has improved PEEK's mechanical properties, making CFR-PEEK a more alluring choice for metallic products. PEEK material is expected to be used in dental post constructions and the field of endodontics in the future due to its superior mechanical and biological qualities. Polymer prostheses will also likely find a place in common applications. Due to properties of PEEK and modification of PEEK it can be potentially used for future developments.
